# Identification of key elements and driving mechanisms in university early warning systems for suicide and violence crises—Based on the TOE framework and the DEMATEL-ISM model

**DOI:** 10.3389/fpubh.2026.1874410

**Published:** 2026-07-17

**Authors:** Haijiang Wang

**Affiliations:** Office of Student Affairs, Zhijiang College of Zhejiang University of Technology, Shaoxing, China

**Keywords:** DEMATEL-ISM, early warning system, psychological crisis, suicide and violence crises, TOE framework

## Abstract

**Purpose:**

This study aims to elucidate the internal operational logic of psychological crisis early warning systems (PCEWS) for college students, overcome the limitations of static and unidimensional perspectives in existing research, and provide theoretical support and practical pathways for system optimization.

**Methods:**

Based on the Technology-Organization-Environment (TOE) framework, 12 key elements of the PCEWS were extracted. Drawing upon the empirical operational records of 13 representative universities and domain expert knowledge, an integrated DEMATEL-ISM (Decision Making Trial and Evaluation Laboratory—Interpretive Structural Modeling) approach was employed to analyze the causal relationships and hierarchical driving mechanisms among these elements.

**Results:**

The PCEWS is comprehensively constrained by multidimensional elements. Financial investment, the formalization of mechanisms, and human resource allocation are identified as the foundational elements, exhibiting the highest centrality and a strong degree of cause. The system exhibits a three-tier hierarchical driving structure: the deep-level financial and institutional elements, through resource injection and institutional empowerment, drive the middle-level data updating processes and systematic training construction. These, in turn, sustain the surface-level outcomes, including the proactiveness of early warnings, cross-departmental collaboration, and the effectiveness of home-school synergy.

**Conclusions:**

This study rectifies the misconception in PCEWS construction that overemphasizes technology at the expense of management. It provides a scientific decision-making basis and optimization pathways for universities to precisely allocate mental health resources and eliminate operational barriers within the early warning system.

## 1 Introduction

Mental health issues among university students have emerged as a global public health challenge. According to reports by the World Health Organization, mental health disorders are among the leading causes of morbidity and disability in adolescents. The persistently high prevalence of depression, anxiety, and suicidal ideation among the university student population constitutes a severe global public health crisis. In China, the detection rate of depression among adolescents has reached 24.6%, with severe depression accounting for 7.4%, indicating a concerning trend of psychological issues occurring at a younger age. Universities, as high-risk environments for psychological crisis events, are facing severe challenges in their mental health operations. Surveys indicate that the prevalence of psychological disorders among university students is 20.23%. Furthermore, the number of students suspending their studies or dropping out due to psychiatric disorders accounts for 37.9% and 64.4% of the total medical suspensions and dropouts, respectively. Against this backdrop, the construction of a highly efficient psychological crisis early warning system (PCEWS) is regarded as the core strategy for transitioning from “passive response” to “proactive prevention” in campus behavioral health management. Consequently, various universities have vigorously promoted the construction of PCEWS; however, numerous dilemmas persist in practical implementation. Traditional early warning methods predominantly rely on scale-based screening and subjective observation, which suffer from limited identification accuracy and delayed responses. With the advancement of information technology, emerging technologies such as big data and artificial intelligence have been gradually applied to the mental health domain, offering new possibilities for enhancing early warning capabilities. While this study focuses on suicide and violence-related crises to maintain analytical clarity, future iterations should extend coverage to acute psychotic episodes and severe mood disturbances to ensure a comprehensive campus public health safety net.

In a broad sense, a psychological crisis refers to a series of psychologically imbalanced reactions that occur when an individual's customary coping resources are insufficient to handle frustration or trauma. Psychological crises do not conform to the general laws of causality; rather, they are subject to the interactive effects of multiple factors, including precipitating events, environments, and internal psychological states, thereby manifesting in diverse, complex, and dynamic forms (1). In alignment with the practicalities of university operations, the “psychological crisis of university students” in this study is specifically delimited to crises of suicide/self-harm and violent behavior toward others. Correspondingly, “psychological crisis early warning” is defined as the behavioral signs indicating that these two types of crises are imminent or currently unfolding. There is a broad consensus among scholars that the majority of crises can be prevented through human intervention if detected in a timely manner. Currently, a major challenge in psychological crisis early warning research lies in the blurred boundary between “risk factors” and “warning signs.” Researchers emphasize that the critical distinction between the two is temporal urgency: warning signs indicate an immediate probability of suicide/self-harm or violence, necessitating emergency intervention; whereas risk factors encompass a range of persistent, long-term characteristics that do not require immediate emergency action (2). Therefore, the “psychological crisis early warning” discussed in this study strictly refers to these imminent warning signs.

## 2 Literature review

Within the Chinese higher education context, research on student psychological crises has long focused on identifying crisis states through assessment tools. For instance, Yang (1) developed a localized University Student Psychological Crisis Screening Scale, while Sun et al. (3) analyzed influencing factors based on the dual-factor model of mental health. Wang et al. (4) and Shu et al. (5) explored predisposing personality traits through the lenses of Traditional Chinese Medicine (TCM) constitutions and borderline personality traits, respectively. Furthermore, Yang et al. (6) proposed an early warning indicator system for individual psychological crisis signs. However, these studies predominantly rely on self-report questionnaires or subjective counselor ratings. They essentially function as “*post-hoc* identification” or “static classification,” failing to meet the demand for dynamic monitoring of the crisis evolution process.

International scholarship advances this discourse by reconstructing psychological crisis not merely as a clinical state, but as a public safety and organizational management issue. Early research emphasized that severe depression and hopelessness are distal predictors of crisis onset (7, 8). However, the literature has since refined the distinction between “risk factors” and “warning signs” (2). Risk factors determine baseline susceptibility and change slowly, whereas warning signs are short-term indicators of impending crisis. This distinction provides a theoretical foundation for building “pre-warning” systems.

The early warning of psychological crises on campus is not a singular assessment step but a layered socio-technical system. International experience outlines its evolution in three stages (9):

Traditional Warning Stage, Reliance on classical scales such as the Beck Scale for Suicide Ideation (BSS) and the PHQ-9. While standardized, these methods are low-frequency, lagging, and limited by students' willingness to seek help (10).

Information System-Assisted Stage, Domestic scholars have attempted to construct early warning models using multi-source data such as attendance, consumption, and dormitory access logs (11–14). However, existing models often remain at the level of “correlation mining” and lack deep consideration of false-positive costs and privacy ethics.

Big Data Integration Stage, Attempts to introduce machine learning algorithms often face practical governance dilemmas such as “data silos” and “model opacity.”

Notably, international studies indicate that an individual's insight often deteriorates just before a crisis erupts; thus, observations from “gatekeepers”—such as peers, dorm supervisors, and counselors—are more critical than self-reports (15). Consequently, an effective early warning system must transcend mere data analysis and focus on organizational response mechanisms.

Given that existing research primarily uses regression analysis to explore “the effect of X on Y,” it struggles to clarify the hierarchical structure and causal relationships among elements. Thus, introducing the Technology-Organization-Environment (TOE) framework is essential. Originally used to explain technological innovation adoption (16), the TOE framework has been widely applied in e-government and emergency management (17, 18).

In the context of campus psychological crisis early warning:

(1) The Technological dimension addresses data mining algorithms and platform integration capabilities.
(2) The Organizational dimension involves departmental coordination, personnel competency, and institutional norms.
(3) The Environmental dimension encompasses policy regulations, campus culture, and social support.

Unlike traditional symmetric causal inference, the failure of a psychological crisis early warning system is often due to the absence of certain Necessary Conditions. As Dul (19) argues in Necessary Condition Analysis (NCA), specific organizational or technical elements are indispensable; without them, the system cannot function regardless of the strength of other factors. Therefore, this study integrates the TOE framework with the DEMATEL-ISM method to reveal the driving relationships and hierarchical structures among various elements, rather than merely verifying correlations.

In summary, while existing studies have identified numerous factors influencing psychological crises, significant gaps remain at the system level:

(1) an overemphasis on technical algorithm optimization while neglecting the constraints of organizational structure and environmental pressure;
(2) a reliance on linear correlation assumptions without revealing the non-linear causal relationships and hierarchical structures among elements. Based on these gaps, this study introduces the TOE framework and applies the DEMATEL-ISM method to analyze the constituent elements and multi-level hierarchical structure of the university psychological crisis early warning system. The aim is to answer the core question: “Which factors are the deep-rooted drivers determining system efficacy, and which are merely superficial symptoms?”

## 3 Method

### 3.1 Subjects and data sources

**Figure 1:**
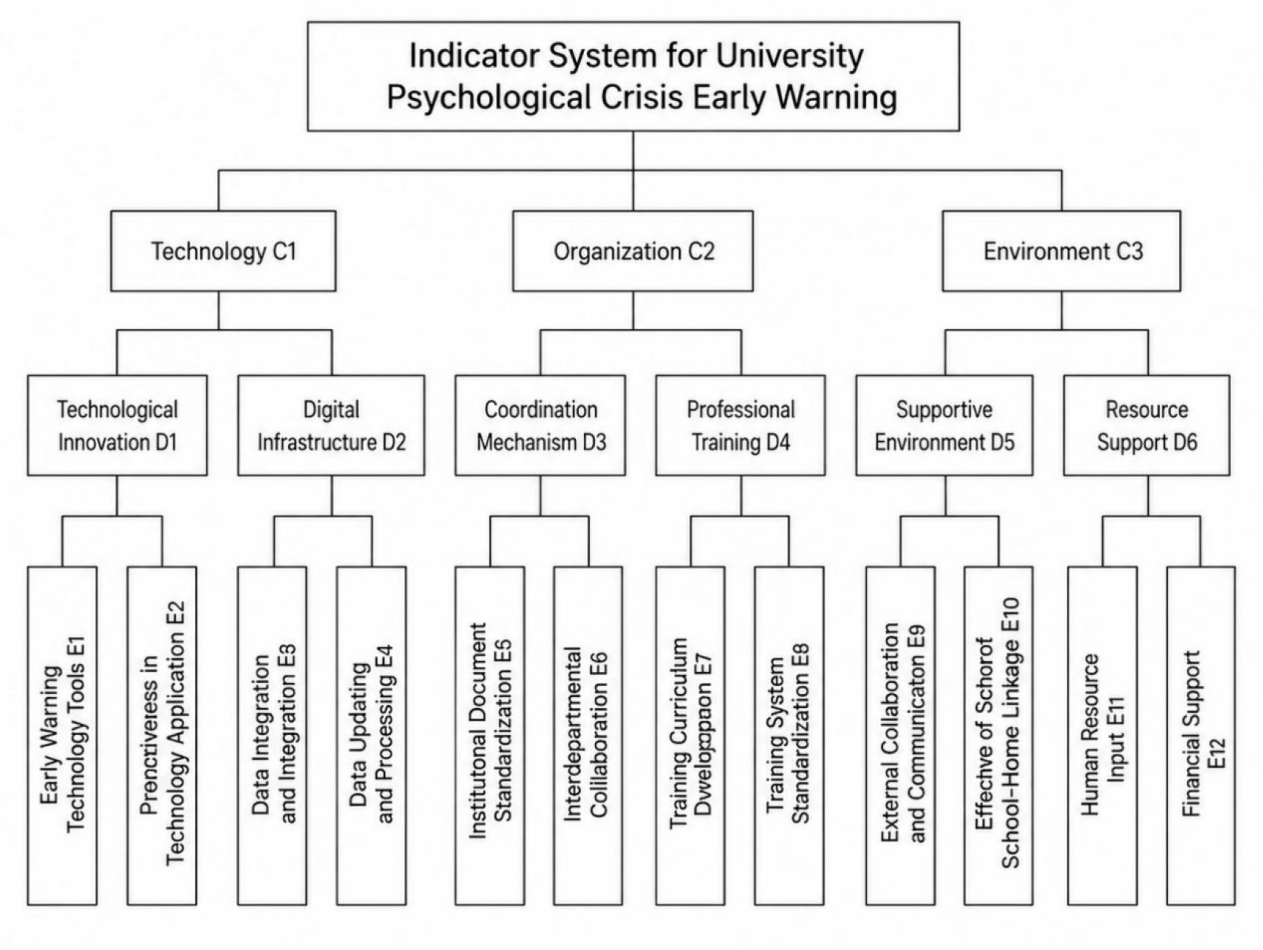
Indicator system university psychological crisis early warning.

**Figure 2:**
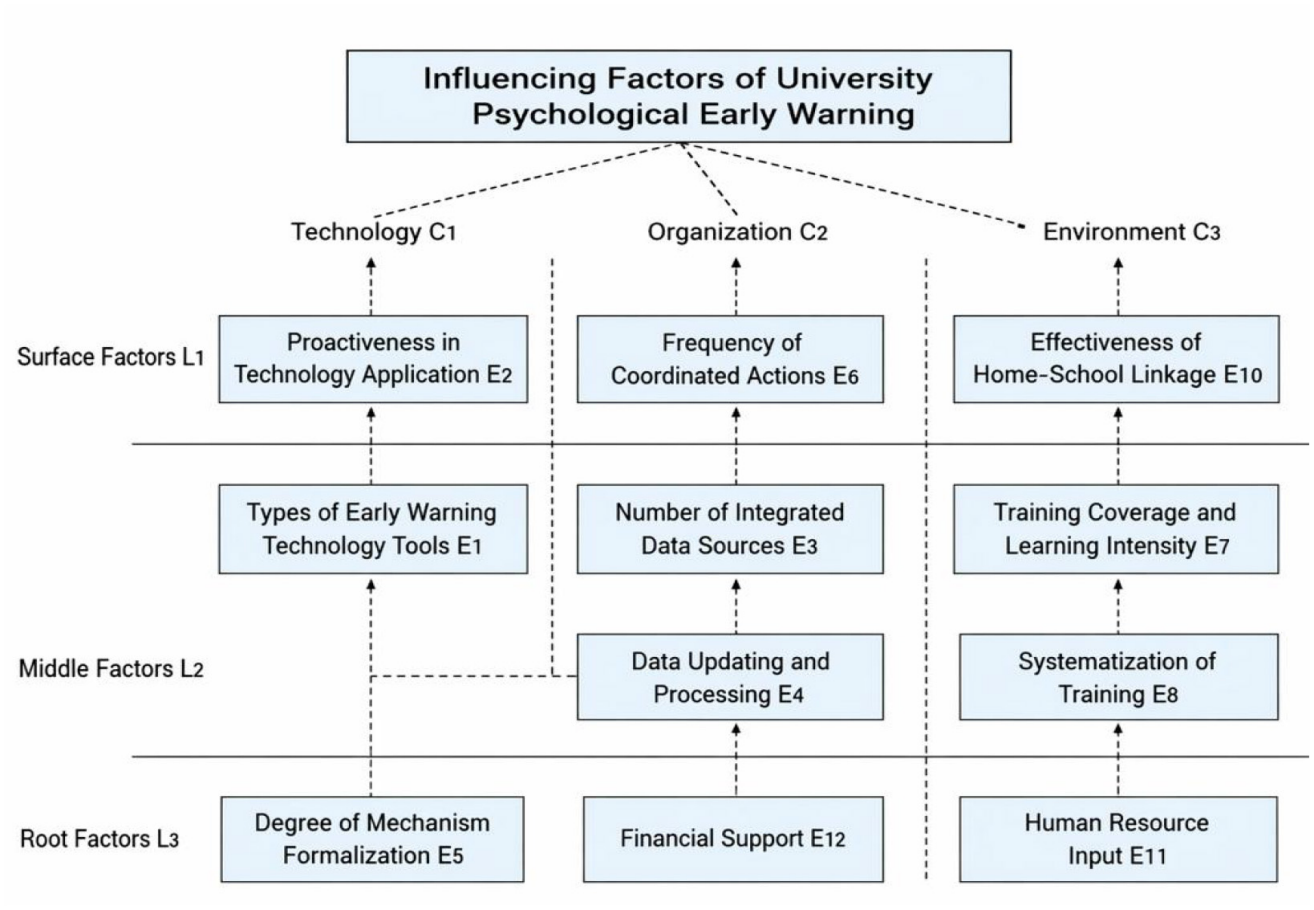
ISM model.

First, based on the Technology-Organization-Environment (TOE) theoretical framework, this study conducted an extensive literature review and policy text analysis to initially extract the potential elements influencing the operation of the psychological crisis early warning system (PCEWS) in universities (Figure 1). To ensure the scientific validity and contextual applicability of the indicators, the research team invited three senior experts in the field of university mental health education to conduct multiple rounds of discussion and refinement on the preliminary indicators. Ultimately, 12 key system elements were established, encompassing the technological dimension (four items), organizational dimension (four items), and environmental dimension (four items; see Figure 2). These 12 elements constitute the network nodes for the subsequent DEMATEL-ISM analysis.

The TOE framework was adopted to categorize and integrate the influencing factors of the university student PCEWS. Proposed by Tornatzky and Fleischer (16), the TOE framework is a comprehensive analytical model suitable for technology adoption contexts. Because this framework does not rigidly pre-define the specific elements within each dimension, it possesses strong flexibility and operability, demonstrating excellent utility in explaining the causes and influencing factors of complex social phenomena. In multimodal contexts, the TOE framework has proven its robust applicability in various complex efficiency-enhancement studies, such as the efficiency of disaster public warnings, the transformation efficiency of university scientific and technological achievements, and the efficiency of local government public opinion governance. By adopting a holistic perspective, the TOE framework comprehensively analyzes the multidimensional impacts of technology, organization, and environment on the PCEWS. It is highly applicable for interpreting the critical context of psychological crisis early warning, facilitating the clarification of the main operational factors, and providing a logical structure for elucidating the subsequent relationships among these elements.

The technological dimension breaks the limitations of the traditional reactive warning paradigms through scientific and technological innovation and digitalization, thereby achieving proactive prediction. On one hand, technological innovation embeds frontier technologies—such as big data, artificial intelligence, and the Internet of Things (IoT)—into the early warning workflow. This replaces or supplements traditional scale screenings and manual observations, enabling the real-time and unobtrusive detection of crisis signals, which significantly shortens the warning response cycle. On the other hand, digitalization dismantles the “data silos” across various university departments. It enables collaborative data processing among the Student Affairs Office, Academic Affairs Office, Logistics Management Department, and others. By cross-analyzing the correlational features of multi-source data, it constructs a comprehensive, 360-degree student persona, precisely locating individuals with hidden psychological crises.

The organizational dimension comprises two sub-elements: collaborative processing and professional training. While organizations are influenced by technology, they simultaneously exert a reciprocal effect on it, reshaping and reconstructing technological resources. In universities, a collaborative network for crisis early warning is formed among the Student Affairs Office, the Mental Health Center, secondary colleges, the Security Office, and the university hospital. A disconnection in any single department can lead to warning failures and irreversible consequences. Furthermore, the institutionalization and normalization of professional training have been proven to play a crucial role. Personnel without systematic training are prone to biases when identifying initial crisis signs. Institutionalized and routine training helps internal members enhance their capabilities in crisis signal identification, communication skills, and preliminary reporting, thereby effectively improving early warning efficiency.

The environmental dimension includes the macro-environment and the intensity of support. The macro-environment in which an organization operates acts as an exogenous factor driving technology application. It not only moderates the dynamic role of internal organizational factors but also provides the necessary conditions for the interaction between the organization and technology. The macro-environment for psychological crisis early warning encompasses families, universities, medical institutions, and communities. For families, effective communication mitigates information asymmetry and secures familial support for interventions. For medical institutions, establishing fast-track referral pathways overcomes the bottleneck caused by universities lacking medical diagnostic authority. For communities, linking with community resources ensures the long-term continuity of crisis interventions. Meanwhile, specific policies and resource support reflect the degree of financial and policy backing from the university's internal administration and higher authorities. Sufficient earmarked funding is the foundation for early warning operations. Furthermore, incorporating mental health warnings into the university's annual strategic objectives and “Safe Campus” evaluation metrics significantly boosts the proactive participation of all departments (Table 1).

**Table 1:** Measurement indicators and descriptions.

Measurement indicator	Indicator description
Types of early warning technical tools	0 = No dedicated tools; 1 = Online standardized psychological scale system; 2 = Basic data dashboards; 3 = Introduction of basic predictive models; 4 = Deployment of advanced predictive models
Proactiveness of technology application	0 = Completely passive response; 1 = Semi-proactive screening; 2 = Proactive early warning
Number of integrated data sources	0 = No integration; 1 = Integrates 1-2 systems; 2 = Integrates 3-4 systems; 3 = Integrates 5 or more systems
Data updating and processing	0 = No real-time data; 1 = Monthly/semester batch import; 2 = Real-time/near real-time (T+1) synchronization of key data; 3 = Real-time processing and calculation of full data streams
Degree of mechanism formalization	0 = No written mechanism; 1 = Informal communication groups or conventions; 2 = Official university-level documents
Frequency of collaborative action	Number of joint case-study seminars held by multiple departments
Coverage and intensity of training	Pass rate of counselors participating in crisis identification training assessments
Systematic training	0 = No regular training; 1 = Sporadic lectures; 2 = Annual training plan with basic execution; 3 = Tiered and classified curriculum system with certification
External protocols and channels	0 = No stable external cooperation; 1 = Green channel protocols with at least one specialized psychiatric hospital; 2 = Official cooperation agreements or designated contacts with at least two types of external entities (e.g., hospitals, communities/sub-districts, public security)
Effectiveness of home-school synergy	Proportion of parents successfully contacted and effectively communicated with in high-risk cases that have occurred
Human resource allocation	Ratio of full-time teachers in the mental health center to the total number of students
Financial support	Per-student earmarked funding for mental health work

In summary, under the TOE framework, the factors influencing the efficiency of university psychological crisis early warning comprehensively encompass three dimensions: technological capability, organizational readiness, and environmental support.

Indicator content validity was ensured through a two-round Delphi procedure with three senior experts. Inter-expert consistency was satisfactory (coefficient of variation < 0.15). Operational definitions for borderline items (e.g., E9, E11, E12) were iteratively refined to align with Chinese university administrative realities.

### 3.2 Methodology

The Decision-making Trial and Evaluation Laboratory (DEMATEL) is a systematic research method based on graph theory and mathematical matrix analysis. Its core function lies in revealing the degree of importance of causal relationships among elements within a complex system. By utilizing expert scoring to evaluate causal relationships, this method progressively constructs a relationship matrix. Through calculating the direct influence matrix and the comprehensive influence matrix, it ultimately derives the degree of influence (R), the degree of being influenced (C), the centrality (D+C), and the degree of cause (D–C) for each factor. This process clarifies the core weights and hierarchical roles of all influencing factors within the entire system.

Interpretative Structural Modeling (ISM) is a classic analytical method in the field of systems engineering. Its primary objective is to disentangle the logical structural connections among system elements. By decomposing the interactive relationships among these elements, it clarifies their hierarchical structure, providing a clear framework for system analysis and optimization. Therefore, the integrated DEMATEL-ISM approach not only reveals the logical relationships among the influencing factors during the operation of the university student psychological crisis early warning system (PCEWS) but also provides robust decision support for university administrators.

The specific mathematical modeling steps are as follows:

Step 1: construct the direct influence matrix.

Experts were invited to evaluate the degree of direct influence between factors, yielding an n × n direct influence matrix A, where *a*_*ij*_ represents the degree to which factor i influences factor j as judged by the experts.


$$\begin{array}{l} A=|a_{ij}{|}_{n\times n} \end{array}$$ (1)

In Equation 1, *a*_*ij*_ is the average value of the influence scores given by the experts. When i = j, *a*_*ij*_ = 0 meaning a factor does not influence itself.

Step 2: normalize the direct influence matrix.

The direct influence matrix A is standardized to obtain the DEMATEL normalized matrix $B$B.


$$\begin{array}{l} B=\frac{A}{\max\;\left(\max\overset{n}{\underset{i=1}{\sum }}a_{ij},\max\overset{n}{\underset{j=1}{\sum }}a_{ij}\right)} \end{array}$$ (2)

In Equation 2, the denominator represents the maximum value between the largest sum of row elements and the largest sum of column elements in matrix $A$.

Step 3: calculate the comprehensive influence matrix.

The DEMATEL comprehensive influence matrix Z is calculated based on the following formula:


$$\begin{array}{l} Z=\overset{\infty }{\underset{i=1}{\sum }}B^{i}=B{\;\left(I-B\right)}^{-1} \end{array}$$ (3)

In Equation 3, where I is the identity matrix.

Step 4: calculate the specific index values.

Based on the comprehensive influence matrix Z, the influence degree (R) and the influenced degree (C) can be calculated.


$$\begin{array}{l} R=\overset{n}{\underset{j=1}{\sum }}Z_{ij},i=1,2,...,n \end{array}$$ (4)

$$\begin{array}{l} C=\overset{n}{\underset{i=1}{\sum }}Z_{ij},i=1,2,...,n \end{array}$$ (5)

In Equations 4 and 5, *Z*_*ij*_ represents the specific numerical value in the comprehensive influence matrix Z. The degree of cause (R–C) indicates the factor type: a positive value denotes a causal factor (cause group), while a negative value denotes an effect factor (effect group). The centrality (R+C) indicates the relative importance of the factor; a larger centrality value signifies greater importance.

Step 5: calculate the reachability matrix.

By incorporating the identity matrix I, the comprehensive influence matrix Z is transformed into the overall influence matrix H (i.e., H=Z+I) to reflect the elements' self-influence. Next, a threshold value λ is set based on matrix H to reflect the looseness of the system structure. To mitigate the subjective bias of expert scoring, the sum of the statistical mean and standard deviation is adopted as the value for λ. Boolean algebraic operations are then applied until the matrix reaches a steady state, yielding the reachability matrix $K$.


$$\begin{array}{l} \lambda =\alpha +\beta ,\lambda \in \left[0,1\right] \end{array}$$ (6)

$$K=\{\begin{array}{c} 1,h_{ij}\geq \lambda \\ 0,h_{ij}<\lambda \end{array}\;i,j=1,2,...,n$$ (7)

In Equations 6 and 7, where α and β represent the mean and standard deviation of all elements in the comprehensive influence matrix Z, respectively, and *h*_*ij*_ represents the specific numerical value in the overall influence matrix $H$.

Step 6: construct the multi-level hierarchical structural model.

The reachability matrix K is decomposed to calculate the reachability set R (*k*_*i*_), the antecedent set Q (*k*_*i*_), and the intersection set S. Hierarchical partitioning is then performed to ultimately construct the multi-level hierarchical structural model of the influencing factors for the university student PCEWS.


$$\begin{array}{lll} R\;\left(k_{i}\right) & = & \left\{k_{i}|k_{ij}=1\right\},i=1,2,3,...,n \end{array}$$ (8)

$$\begin{array}{lll} Q\;\left(k_{i}\right) & = & \left\{k_{i}|k_{ji}=1\right\},i=1,2,3,...,n \end{array}$$ (9)

$$\begin{array}{lll} R\;\left(k_{i}\right) & ⋂ & Q\;\left(k_{i}\right)=S \end{array}$$ (10)

In Equations 8–10, the reachability set R (*k*_*i*_) represents the set of all elements with a value of 1 in a specific row of the reachability matrix, while the antecedent set R (*k*_*i*_) represents the set of all elements with a value of 1 in a specific column.

### 3.3 Data processing and hybrid evaluation

Traditional DEMATEL methods rely heavily on subjective expert scoring, making them susceptible to cognitive bias. To enhance the objectivity of the direct influence matrix, this study adopted a hybrid evaluation approach that integrates objective empirical data analysis with domain expert calibration. The sample comprises 13 public universities in Zhejiang Province, stratified by institutional type (comprehensive, engineering, normal, and vocational). The data cover the period 2019–2024 and include 12 operational variables per institution. Missing values (< 5%) were imputed using expectation maximization. The sample represents approximately 18% of provincial undergraduate enrollments, balancing geographic distribution and institutional scale. By analyzing the partial correlation trends within these panel data, a preliminary relational baseline among the elements was constructed. Specifically, partial correlation coefficients were first calculated from the panel data to establish a baseline adjacency structure among the 12 elements. This empirical baseline was then presented to the expert panel to calibrate direct influence intensities, reducing the risk of purely subjective anchoring.

Subsequently, an expert panel was convened, comprising five directors of University Student Affairs Offices and directors of University Mental Health Centers. Based on the objective empirical baseline derived from the 13 universities, the experts conducted independent scoring to evaluate the degree of direct influence among the 12 elements. The evaluation utilized a standard 0–4 scale (0 = no influence, 1 = weak influence, 2 = moderate influence, 3 = strong influence, 4 = extreme influence). Finally, the arithmetic mean of the experts' independent scores was computed to generate the initial direct influence matrix $A$.

## 4 Results

### 4.1 Identification of key influencing factors based on DEMATEL

It should be emphasized that all causal attributions in this study refer strictly to “modeled influence relationships” derived from DEMATEL-ISM logic, rather than empirically verified causal effects in a counterfactual sense. To elucidate the causal relationships and relative importance of the various elements within the university psychological crisis early warning system (PCEWS), this study employed the DEMATEL method to calculate the influence degree (R), influenced degree (C), centrality (R+C), and degree of cause (R–C) for the 12 key variables. Centrality reflects the overall importance of a specific factor within the entire early warning system. A degree of cause greater than zero (R–C > 0) indicates that the variable belongs to the cause group (i.e., a causal factor that actively influences other factors), whereas a value less than zero (R–C < 0) classifies it into the effect group (i.e., a result factor predominantly influenced by other factors). The detailed calculation results are presented in Table 2.

**Table 2:** The DEMATEL calculation results of the influencing factors for the PCEWS.

Dimension	Variables	Influence degree (R)	Influenced degree (C)	Centrality (R+C)	Degree of cause (R-C)	Type
Technological	E1	1.108	1.573	2.681	−0.465	Effect
	E2	0.979	1.828	2.807	−0.849	Effect
	E3	1.246	1.632	2.878	−0.386	Effect
	E4	1.117	1.703	2.820	−0.586	Effect
Organizational	E5	2.051	1.368	3.419	0.683	Cause
	E6	1.187	2.096	3.283	−0.909	Effect
	E7	1.464	1.342	2.806	0.121	Cause
	E8	1.653	1.343	2.996	0.310	Cause
Environmental	E9	1.264	1.259	2.523	0.005	Cause
	E10	0.955	1.652	2.607	−0.697	Effect
	E11	2.364	1.051	3.415	1.313	Cause
	E12	2.233	0.775	3.008	1.458	Cause

### 4.2 Analysis of key influencing factors based on DEMATEL results

Based on the detailed calculation results (as shown in Table 2), regarding centrality (R+C), the degree of mechanism formalization (E5), human resource allocation (E11), the frequency of cross-departmental collaboration (E6), financial support (E12), and systematic training construction (E8) rank as the top five among the 12 factors. This indicates their critical position within the operation of the university student psychological crisis early warning system (PCEWS) and their tight connections with other elements. These five factors span the organizational and environmental dimensions, demonstrating that the efficacy of the PCEWS is predominantly driven by institutional norms, human and financial resources, and inter-departmental coordination.

Specifically, the centrality of mechanism formalization (E5, organizational dimension) and human resource allocation (E11, environmental dimension) rank at the very top (3.419 and 3.415, respectively). This highlights that top-level institutional design and adequate staffing play a decisive role in system construction. Notably, the influenced degree (C) of E6 (cross-departmental collaboration) is as high as 2.096 (ranking second overall), suggesting that E6 is a strong receiver in the network. Although E6 possesses high centrality (3.283, third highest), its negative degree of cause (R–C = −0.909) classifies it as an effect factor. That is, E6 is strongly influenced by upstream cause factors such as E5, E11, and E12 rather than driving them. Therefore, to promote the highly efficient operation of the system, it is urgently required to strengthen those foundational cause factors that constrain the performance of key effect nodes like E6.

Furthermore, the influence degrees (R) of human resource allocation (E11), financial support (E12), and mechanism formalization (E5) rank in the top three (2.364, 2.233, and 2.051, respectively). This demonstrates that personnel and financial guarantees (environmental dimension) and institutional norms (organizational dimension) exert the strongest impact on the remaining elements. Looking at the degree of cause (R–C), financial support (E12) scores the highest (1.458), followed by human resource allocation (E11, 1.313) and mechanism formalization (E5, 0.683). This reveals that macro-level funding allocation, sufficient staffing, and top-level institutional design serve as the fundamental wellspring driving the entire early warning system.

Based on the positive and negative attributes of the degree of cause (R–C), all influencing factors can be categorized into a cause group and an effect group. Within the cause group (R–C > 0), financial support (E12), human resource allocation (E11), mechanism formalization (E5), systematic training construction (E8), and external protocols and channels (E9) rank in the top five (note: E9 has R–C = 0.005, marginally positive). This identifies them as the foundational drivers operating the PCEWS. From a dimensional perspective: environmentally, the efficient supply of financial and human resources acts as the core driving force of the system. Organizationally, mechanism formalization and systematic training institutionally standardize the intervention behaviors of internal subjects (e.g., full-time psychological teachers and counselors). Externally, protocols and channels (E9) facilitate linkages with medical and social resources, though its near-zero R–C value indicates a balanced role.

Among the effect group factors (R–C < 0), the frequency of cross-departmental collaboration (E6), the proactiveness of technology application (E2), the effectiveness of home-school synergy (E10), and data updating and processing (E4) exhibit the highest absolute values (−0.909, −0.849, −0.697, and −0.586, respectively). This indicates that these factors are the direct practical manifestations of deep-level resource inputs. Organizationally, the frequency of cross-departmental collaboration (E6) is the most prominent effect factor, reflecting how institutional norms and resource allocation translate into operational practice. Technologically, the proactiveness of technology application (E2) and data updating and processing (E4) rely heavily on upstream cause factors such as financial support and training. Environmentally, the effectiveness of home-school synergy (E10) is a surface-level output of crisis interventions in specific contexts. The significant improvement of these effect factors ultimately signifies the successful realization of the PCEWS's efficacy.

### 4.3 Hierarchical structure partitioning of the system based on ISM

To further clarify the hierarchical progressive relationships among the 12 elements, this study introduced a threshold λ based on the DEMATEL comprehensive influence matrix to filter out weak influence relations and establish the reachability matrix. Through the hierarchical extraction algorithm, the interpretative structural model of the influencing factors for the university psychological crisis early warning system (PCEWS) was obtained (as shown in Figure 2). By extracting and integrating the independent factors in each row, a three-level hierarchical structure was constructed (Figure 2).

As clearly observed in Figure 2, the degree of mechanism formalization (E5), financial support (E12), and human resource allocation (E11) are the root elements (L3) driving the system's operation. This indicates that the construction of the PCEWS is not an ungrounded concept; its underlying logic heavily relies on top-level institutional design and basic resource guarantees. Financial support and human resource allocation serve as the fundamental lifeblood of early warning operations. Abundant per-student funding directly drives the construction and iteration of the underlying data processing architecture. Meanwhile, the human resource guarantee of a full-time psychological teacher team is the absolute prerequisite for conducting any crisis intervention and organizational training. Furthermore, mechanism formalization directly provides legitimate authorization and institutional guidance for the procurement, deployment, and implementation of early warning technological tools through official institutional documents and regulations. Through “resource injection” and “institutional standardization,” these three factors jointly construct the solid foundation of the university psychological crisis defense network.

The intermediate-level factors (L2) serve as intermediary elements connecting the surface-level manifestations with the deep-level foundation, possessing a pivotal transitional characteristic. Figure 2 shows that L2 involves the type of early warning technological tools (E1), the number of integrated data sources (E3), data updating and processing (E4), the coverage and intensity of training (E7), and systematic training construction (E8). This indicates that foundational static resources must be transformed into dynamic “digital computational power” and “personnel professional competence” at this level. In the technological transmission pathway, data updating and processing (E4) plays a core driving role. It not only upwardly supports the breadth of multi-departmental data integration (E3) but also directly determines the intelligent evolution degree of technological tools (E1). This implies that real-time, comprehensive data circulation is the prerequisite for breaking information silos and upgrading predictive algorithms. In the organizational transmission pathway, systematic training (E8) inherits the underlying human resource input and effectively translates it into specific training coverage and intensity (E7). This means that an increase in personnel quantity must rely on a tiered and classified systematic curriculum to be genuinely converted into the crisis-identification hard skills of the counselor team. By eliminating technological barriers among heterogeneous systems and closing the professional capability gaps of personnel, these intermediary elements effectively reduce the frictional costs of system operation.

The surface-level factors (L1) include the proactiveness of technology application (E2), the frequency of cross-departmental collaboration (E6), and the effectiveness of home-school synergy (E10). During the actual operation of the PCEWS, these elements are the most direct manifestations of early warning efficacy, representing the ultimate output of intervention behaviors. Specifically, tracing the causal chains indicated by the arrows reveals that: advanced early warning technological tools (E1) directly endow the grassroots system with “forward-deployed computational power,” thereby driving technology application to shift from a passive response to proactive screening (E2). Furthermore, extensive data source integration (E3), by streamlining the operational workflows among student affairs, academic affairs, and logistics, directly boosts the frequency of cross-departmental collaborative judgments (E6). Meanwhile, high-intensity and broadly covered professional training (E7) profoundly empowers front-line educational personnel, significantly enhancing the communication quality and intervention effectiveness of home-school synergy in high-risk cases (E10). Ultimately, these elements form a clear, bottom-up, multi-level coupled pathway, jointly driving the smart reconstruction of the university PCEWS from “passive defense” to “precise identification and highly efficient collaboration.”

## 5 Discussion

### 5.1 Theoretical contributions

First, this study innovatively integrates the TOE framework from organizational management with the DEMATEL-ISM model from systems engineering, applying them across disciplines to the research of university student psychological crisis early warning systems (PCEWS). This cross-boundary application effectively expands the system management boundaries of mental health interventions. Second, this study debunks the common myth of “technological omnipotence” frequently observed in the digital health domain. The research substantiates that advanced early warning technological tools and proactive screening capabilities are merely the surface-level outputs and intermediate transfers of the system. In contrast, financial investments and human resource guarantees (environmental dimension), alongside mechanism formalization (organizational dimension), constitute the underlying foundations that drive the operation of the entire early warning system. This finding significantly deepens the academic community's theoretical understanding of Socio-Technical Systems (STS) alignment. It emphatically underscores that in psychological crisis interventions—which are characterized by high concealment and suddenness—top-level institutional regulations and fundamental resource supplies possess an unsurpassable foundational primacy.

### 5.2 Practical implications

This study provides a clear intervention blueprint for university administrators to construct and optimize the PCEWS. Administrators should allocate resources in accordance with the bottom-up hierarchical evolutionary rule.

First, consolidate the underlying foundation. University party committees and administrative departments must establish multi-departmental collaborative early warning mechanisms through official institutional documents. Furthermore, they must rigorously guarantee the earmarked per-student mental health funding and the appropriate ratio of full-time psychological teachers. These elements serve as the essential “cornerstones” preventing the collapse of the defense line.

Second, unblock the intermediate hubs. Once foundational resources are secured, administrators should avoid the blind pursuit of advanced predictive algorithms. Instead, funding should be prioritized to dismantle data silos among student affairs, academic affairs, and security departments, thereby achieving comprehensive and real-time data updates. Simultaneously, a tiered and classified counselor training system must be established to translate sheer human resource quantities into professional crisis-identification capabilities.

Third, unleash surface-level efficacy. Building upon the aforementioned steps, grassroots execution departments can genuinely realize the transition of technology application from a “passive response” to a “proactive strike.” Furthermore, relying on previously established fast-track green channel protocols, they can achieve highly efficient home-school-medical synergy and closed-loop management when high-risk cases occur.

### 5.3 Limitations and future directions

#### 5.3.1 Methodological constraints

False positives pose a non-trivial clinical risk: unnecessary alerts may induce iatrogenic anxiety, damage trust, or trigger coercive interventions. Accordingly, system outputs are explicitly positioned as preliminary risk signals rather than clinical diagnoses, and must be validated through tiered clinical review prior to any formal action.

To translate algorithmic alerts into actionable clinical assessments without violating confidentiality or triggering premature coercive measures, this study proposes adopting the Gatekeeper Training Model. The tiered clinical review process is defined as follows:

Level 1 (Peer/Gatekeeper Observation): initial alerts trigger enhanced observational protocols by trained non-medical staff rather than immediate punitive or compulsory actions.

Level 2 (Clinical Triage): only cases exhibiting sustained high-risk signals are escalated to licensed mental health professionals for formal clinical assessment. This staged approach ensures that digital warnings serve as prompts for human-centered care rather than automated sanctions, effectively mitigating the risks of iatrogenic harm and stigma identified in Gould et al. (9).

This study acknowledges certain limitations that warrant further exploration. Methodologically, the construction of the direct influence matrix in the DEMATEL-ISM model inevitably relies on the knowledge judgments of domain experts. Although this study innovatively introduced objective panel data from 13 representative universities as an evaluation baseline to mitigate subjective bias, the limited number of experts and their inherent cognitive boundaries may still exert a certain impact on the model's ultimate precision. Furthermore, this study primarily constructed a static topological structure of the university PCEWS, which fails to depict the dynamic evolutionary characteristics of the system. Future research could integrate the System Dynamics (SD) methodology to simulate optimal evolutionary strategies under dynamic resource allocation conditions.

Beyond methodological constraints, several critical clinical and ethical boundaries must be explicitly addressed. First, regarding data privacy and the early warning technology tools (E1), integrating cross-departmental data poses significant risks of digital surveillance. Future system implementations must strictly adhere to data anonymization protocols and secure explicit informed consent from students to prevent stigmatization. Second, a highly efficient algorithmic warning system does not equate to clinical accuracy. To mitigate the profound psychological harm caused by algorithmic false-positives, the system's alerts must serve only as a preliminary screening mechanism, seamlessly handing over to licensed psychiatrists or clinical psychologists for definitive diagnosis; administrative staff should never cross clinical boundaries. Third, the Effectiveness of home-school synergy (E10) mechanism must be executed with extreme clinical caution. In cases where the family environment is the source of trauma, automatic parental notification could catastrophically exacerbate the student's distress, thus requiring a mandatory “harm-potential assessment” by mental health professionals prior to any family involvement. Finally, this study predominantly conceptualizes psychological crises around acute high-risk behaviors (e.g., suicide and violence). Future research must broaden this definition to accommodate other severe psychiatric emergencies, such as acute psychosis or severe manic episodes, ensuring a holistic campus public health safety net.

#### 5.3.2 Ethical considerations and clinical boundaries

This study does not involve individually identifiable student data. The dataset used for analysis consists exclusively of aggregated institutional-level operational indicators collected over 5 years. As such, this research constitutes a non-human subjects study as defined by the Institutional Review Board, and the requirement for individual informed consent is waived under the exemption for aggregated operational research.

Beyond these methodological and regulatory considerations, we emphasize that the operational deployment of a real-world PCEWS would require strict ethical and clinical boundaries. In such a deployment, the “360-degree student profile” would be constructed under the principle of data minimization. Explicit informed consent would be obtained for any behavioral data beyond academic records, and students would retain the right to opt out of non-core data streams. Administrative and counseling staff would operate strictly within non-clinical boundaries. Definitive risk adjudication and crisis response decisions would be reserved for licensed psychiatrists or clinical psychologists; non-clinical personnel could initiate referral protocols but not diagnostic judgments. Home-school synergy protocols would mandate a mandatory “harm-potential assessment”: if the family environment were identified as a primary trauma source, parental notification would be contraindicated unless mediated by clinical professionals.

In summary, the present study itself is exempt from individual consent requirements due to its use of aggregated institutional data; however, the ethical safeguards described above represent the standards that should govern any future implementation of the proposed system.

## Data Availability

The raw data supporting the conclusions of this article will be made available by the authors, without undue reservation.
