# Supplementary material for: Identification of key elements and driving mechanisms in university early warning systems for suicide and violence crises—Based on the TOE framework and the DEMATEL-ISM model
Source: Front Public Health. 2026 Jul 17;14:1874410. doi: 10.3389/fpubh.2026.1874410 (PMC13423709; doi:10.3389/fpubh.2026.1874410)
Supplement: Supplementary file 1 [file Supplementary_File_1.docx]

**Supplementary Appendix**

**Relation matrix**

|  | E1 | E2 | E3 | E4 | E5 | E6 | E7 | E8 | E9 | E10 | E11 | E12 |
| --- | --- | --- | --- | --- | --- | --- | --- | --- | --- | --- | --- | --- |
| E1 | 0 | 3 | 2 | 3 | 1 | 2 | 1 | 1 | 1 | 2 | 1 | 1 |
| E2 | 2 | 0 | 2 | 2 | 1 | 3 | 1 | 1 | 1 | 2 | 1 | 0 |
| E3 | 3 | 2 | 0 | 4 | 2 | 3 | 1 | 1 | 1 | 1 | 1 | 1 |
| E4 | 2 | 3 | 3 | 0 | 1 | 3 | 1 | 1 | 1 | 1 | 1 | 1 |
| E5 | 3 | 3 | 3 | 3 | 0 | 4 | 3 | 4 | 3 | 3 | 2 | 2 |
| E6 | 2 | 2 | 2 | 2 | 2 | 0 | 1 | 1 | 2 | 3 | 1 | 1 |
| E7 | 2 | 3 | 2 | 2 | 2 | 3 | 0 | 3 | 1 | 2 | 2 | 1 |
| E8 | 2 | 2 | 2 | 2 | 3 | 3 | 4 | 0 | 2 | 3 | 2 | 1 |
| E9 | 1 | 2 | 2 | 1 | 2 | 3 | 2 | 2 | 0 | 3 | 1 | 1 |
| E10 | 1 | 2 | 1 | 1 | 1 | 3 | 1 | 1 | 2 | 0 | 1 | 1 |
| E11 | 3 | 4 | 3 | 3 | 4 | 4 | 4 | 4 | 3 | 4 | 0 | 2 |
| E12 | 4 | 3 | 4 | 4 | 3 | 2 | 3 | 3 | 3 | 2 | 4 | 0 |

| **Direct relation matrix** | | | | | | | | | | | | |
| --- | --- | --- | --- | --- | --- | --- | --- | --- | --- | --- | --- | --- |
|  | E1 | E2 | E3 | E4 | E5 | E6 | E7 | E8 | E9 | E10 | E11 | E12 |
| E1 | 0.000 | 0.079 | 0.053 | 0.079 | 0.026 | 0.053 | 0.026 | 0.026 | 0.026 | 0.053 | 0.026 | 0.026 |
| E2 | 0.053 | 0.000 | 0.053 | 0.053 | 0.026 | 0.079 | 0.026 | 0.026 | 0.026 | 0.053 | 0.026 | 0.000 |
| E3 | 0.079 | 0.053 | 0.000 | 0.105 | 0.053 | 0.079 | 0.026 | 0.026 | 0.026 | 0.026 | 0.026 | 0.026 |
| E4 | 0.053 | 0.079 | 0.079 | 0.000 | 0.026 | 0.079 | 0.026 | 0.026 | 0.026 | 0.026 | 0.026 | 0.026 |
| E5 | 0.079 | 0.079 | 0.079 | 0.079 | 0.000 | 0.105 | 0.079 | 0.105 | 0.079 | 0.079 | 0.053 | 0.053 |
| E6 | 0.053 | 0.053 | 0.053 | 0.053 | 0.053 | 0.000 | 0.026 | 0.026 | 0.053 | 0.079 | 0.026 | 0.026 |
| E7 | 0.053 | 0.079 | 0.053 | 0.053 | 0.053 | 0.079 | 0.000 | 0.079 | 0.026 | 0.053 | 0.053 | 0.026 |
| E8 | 0.053 | 0.053 | 0.053 | 0.053 | 0.079 | 0.079 | 0.105 | 0.000 | 0.053 | 0.079 | 0.053 | 0.026 |
| E9 | 0.026 | 0.053 | 0.053 | 0.026 | 0.053 | 0.079 | 0.053 | 0.053 | 0.000 | 0.079 | 0.026 | 0.026 |
| E10 | 0.026 | 0.053 | 0.026 | 0.026 | 0.026 | 0.079 | 0.026 | 0.026 | 0.053 | 0.000 | 0.026 | 0.026 |
| E11 | 0.079 | 0.105 | 0.079 | 0.079 | 0.105 | 0.105 | 0.105 | 0.105 | 0.079 | 0.105 | 0.000 | 0.053 |
| E12 | 0.105 | 0.079 | 0.105 | 0.105 | 0.079 | 0.053 | 0.079 | 0.079 | 0.079 | 0.053 | 0.105 | 0.000 |

| **Total relation matrix T** | | | | | | | | | | | | |
| --- | --- | --- | --- | --- | --- | --- | --- | --- | --- | --- | --- | --- |
|  |  | E1 | E2 | E3 | E4 | E5 | E6 | E7 | E8 | E9 | E10 | E11 |
| E1 | 0.060 | 0.144 | 0.112 | 0.139 | 0.075 | 0.130 | 0.073 | 0.073 | 0.072 | 0.111 | 0.064 | 0.054 |
| E2 | 0.102 | 0.061 | 0.104 | 0.107 | 0.070 | 0.145 | 0.067 | 0.067 | 0.067 | 0.105 | 0.058 | 0.027 |
| E3 | 0.142 | 0.130 | 0.072 | 0.172 | 0.106 | 0.164 | 0.079 | 0.080 | 0.078 | 0.095 | 0.069 | 0.059 |
| E4 | 0.111 | 0.144 | 0.137 | 0.068 | 0.077 | 0.154 | 0.073 | 0.074 | 0.072 | 0.088 | 0.064 | 0.054 |
| E5 | 0.184 | 0.202 | 0.188 | 0.193 | 0.097 | 0.246 | 0.168 | 0.190 | 0.162 | 0.192 | 0.124 | 0.104 |
| E6 | 0.113 | 0.124 | 0.116 | 0.119 | 0.104 | 0.087 | 0.078 | 0.079 | 0.101 | 0.141 | 0.067 | 0.057 |
| E7 | 0.130 | 0.166 | 0.132 | 0.136 | 0.119 | 0.181 | 0.068 | 0.141 | 0.090 | 0.135 | 0.102 | 0.065 |
| E8 | 0.139 | 0.155 | 0.142 | 0.146 | 0.152 | 0.195 | 0.174 | 0.078 | 0.122 | 0.170 | 0.109 | 0.071 |
| E9 | 0.094 | 0.128 | 0.119 | 0.099 | 0.109 | 0.166 | 0.107 | 0.108 | 0.055 | 0.147 | 0.071 | 0.059 |
| E10 | 0.077 | 0.109 | 0.079 | 0.081 | 0.071 | 0.143 | 0.069 | 0.069 | 0.092 | 0.056 | 0.059 | 0.051 |
| E11 | 0.201 | 0.246 | 0.206 | 0.211 | 0.208 | 0.270 | 0.207 | 0.207 | 0.177 | 0.234 | 0.085 | 0.113 |
| E12 | 0.221 | 0.217 | 0.225 | 0.232 | 0.180 | 0.214 | 0.178 | 0.178 | 0.170 | 0.178 | 0.178 | 0.061 |
